# Chemical and Biological Characteristics of Antimicrobial α-Helical Peptides Found in Solitary Wasp Venoms and Their Interactions with Model Membranes

**DOI:** 10.3390/toxins11100559

**Published:** 2019-09-24

**Authors:** Marcia Perez dos Santos Cabrera, Marisa Rangel, João Ruggiero Neto, Katsuhiro Konno

**Affiliations:** 1 Department of Physics, IBILCE, São Paulo State University, São José do Rio Preto, SP 15054-000, Brazil; cabrera.marcia@gmail.com (M.P.d.S.C.); joao.ruggiero@unesp.br (J.R.N.); 2 Immunopathology Laboratory, Butantan Institute, Sao Paulo, SP 05503-900, Brazil; marisarangel2112@gmail.com; 3 Institute of Natural Medicine, University of Toyama, 2630 Sugitani, Toyama, Toyama 930-0194, Japan

**Keywords:** solitary wasp, venom, antimicrobial peptide, linear cationic α-helical peptide, amphipathic α-helix structure, channel-like pore-forming activity

## Abstract

Solitary wasps use their stinging venoms for paralyzing insect or spider prey and feeding them to their larvae. We have surveyed bioactive substances in solitary wasp venoms, and found antimicrobial peptides together with some other bioactive peptides. Eumenine mastoparan-AF (EMP-AF) was the first to be found from the venom of the solitary eumenine wasp *Anterhynchium flavomarginatum micado*, showing antimicrobial, histamine-releasing, and hemolytic activities, and adopting an α-helical secondary structure under appropriate conditions. Further survey of solitary wasp venom components revealed that eumenine wasp venoms contained such antimicrobial α-helical peptides as the major peptide component. This review summarizes the results obtained from the studies of these peptides in solitary wasp venoms and some analogs from the viewpoint of (1) chemical and biological characterization; (2) physicochemical properties and secondary structure; and (3) channel-like pore-forming properties.

## 1. Introduction

Antimicrobial peptides are widely found in plants, insects, amphibians, and mammals, playing an important role in innate immune systems and host defense mechanisms [1,2]. They have attracted much attention as a novel class of antibiotics, in particular for antibiotic-resistant pathogens, because of their action mechanism of non-selective interaction with cell surface membranes of microbes [3,4]. In some cases, antimicrobial peptides are produced when challenged by microbes, and they are also contained in arthropod venoms. They may play a role in preventing potential infection [5,6,7].

We have surveyed bioactive substances in solitary wasp venoms, in particular neurotoxins, because the venom is used for paralyzing their prey. Consequently, we indeed isolated novel peptide neurotoxins, but also found novel antimicrobial peptides. Eumenine mastoparan-AF (EMP-AF) was the first antimicrobial peptide to be found in solitary wasp venoms in the year 2000 [8]. Since then, several other antimicrobial peptides have been found, mostly in solitary eumenine wasp venoms [9,10,11,12,13,14]. These peptides are only 10–15 amino acids in length, are rich in hydrophobic and basic amino acids with no disulfide bond, and can adopt an α-helical amphipathic secondary structure under appropriate conditions. Besides the antimicrobial activity, they commonly show histamine-release from mast cells, as well as hemolytic and leishmanicidal activities. Physicochemical properties of these peptides, i.e., charge, hydrophobicity, and amphipathicity, have been investigated, and some of these properties were shown to be important to our understanding of the structure-function relationship and useful for the design of new sequences with improved biological properties [10,11,12,13,14,15]. The biological activities of these peptides may be due to the adoption of an amphipathic α-helical secondary structure that inserts into the lipids of biological membranes. Accordingly, these peptides were tested in artificial lipid bilayers, and the channel-like activity observed demonstrated that ion conduction through biological membranes must be important to their lytic activity against mammal cells, but more importantly, against microorganisms [10,12,14,15,16]. This review summarizes these results from the studies on antimicrobial α-helical peptides in solitary wasp venoms. An overview of peptide toxins in solitary wasp venoms was summarized previously [17].

## 2. Chemical and Biological Characterization

Chemical studies on solitary wasp venom components were not well documented until we started our own research on solitary wasp venoms in 1995. This may be a result of their solitary lifestyle: collecting a large number of wasp individuals and providing a sufficient amount of venom constituents required for chemical analysis is very difficult. Pioneering studies in 1980s, however, reported on neurotoxins: megascoliakinins in scoliid wasps [18] and philanthotoxins in digger wasps [19]. We collected 40 species of solitary wasps inhabiting Japan, and surveyed bioactive substances in their venoms focused on small molecules and peptides. The peptide neurotoxins, pompilidotoxins (PMTXs), blocking sodium channel inactivation were found first [20], and further survey led to finding antimicrobial peptides, mostly from eumenine wasp venoms. Table 1 summarizes the amino acid sequences of antimicrobial α-helical peptides hitherto found in solitary wasp venoms, and Table 2 shows selected biological activities shown by these peptides.

### 2.1. Mastoparans

Eumenine mastoparan-AF (EMP-AF) was the first antimicrobial α-helical peptide that we found in the venom of the solitary eumenine wasp *Anterhynchium flavomarginatum micado* in the year 2000 [8]. The HPLC profile of the crude venom extracts was rather simple, and accordingly, only one single fractionation led to the purification and isolation of this major peptide. The structure of EMP-AF is homologous to mastoparans found in social wasp venoms (hornets and paper wasps). Mastoparan was originally found in the venom of the social vespid wasp *Vespula lewisii* as a new mast cell degranulating peptide [21]. Since then, many similar peptides have been found in the vespid wasp venoms, and they are collectively called mastoparans or mastoparan peptides [22]. These peptides have common chemical features: they are 14 amino acids in length with C-terminal amidation, they are rich in hydrophobic and basic amino acids, and adapt an α-helical secondary structure under appropriate conditions. Mastoparans exhibit antimicrobial, hemolytic, and mast cell degranulation activities, which are based on this amphipathic chemical character associating with cell membranes [23]. EMP-AF has all the chemical and biological characteristics of mastoparans: in particular, this peptide showed high content of α-helical conformation in 2,2,2-trifluoroethanol (TFE) and sodium dodecylsulfate (SDS) micelles [14], and stimulated degranulation from rat peritoneal mast cells and RBL-2H3 cells to a similar extent as mastoparan [8]. The C-terminal amidation is very important for these chemical and biological properties. For example, a synthetic analog bearing a free-carboxyl terminus (not amidated) showed reduced α-helical content and antibacterial activity [14]. NMR analysis demonstrated that the C-terminal amide contributed to stabilizing the α-helical conformation [24].

Mastoparan-like peptides are commonly distributed in eumenine wasp venoms. EMP-OD (OdVP1) and OdVP3 were found in the venom of the eumenine wasp *Orancistrocerus drewseni drewseni* [25,26]. Despite poor sequence similarity to mastoparan, these peptides have typical chemical features of mastoparans: they are 14 amino acids in length, with an amidated C-terminus, and a possible α-helical secondary structure. EMP-OD exhibits more potent hemolytic activity than that of mastoparan, and OdVP3 shows potent antifungal activity rather than antibacterial activity. EMP-EF and EMP-ER from *Eumenes fraterculus* and *Eumenes rubrofemoratus*, respectively, contain an aspartic acid residue at the second position, which is characteristic for these peptide structures, and less usual for mastoparan peptides. Biological activities (mast cell degranulation, antimicrobial and hemolytic activity) of these peptides are significantly lower than found for mastoparan and EMP-AF [12]. EpVP2a and EpVP2b from *Eumenes pomiformis* are structurally quite similar to EMP-EF and EMP-ER. However, they were found by transcriptomic analysis, and the biological activities were not investigated [27]. EMP-EM1 and EMP-EM2 from *Eumenes micado* are the most recently found mastoparan peptides, and highly homologous to EMP-AF [13]. They show antimicrobial and mast cell degranulating activities at similar extent as EMP-AF, but virtually no hemolytic activity.

### 2.2. Eumenitins

Eumenitin, in the venom of the solitary eumenine wasp *Eumenes rubronotatus*, has basically the same chemical features as those of mastoparans, but an extra hydrophilic amino acid at the C-terminus without amide modification [10]. Accordingly, it rather belongs to linear cationic α-helical peptides. The biological properties are also similar to mastoparans, but the potencies are lower [10]. Three other eumenine wasp venoms contain eumenitin-type peptides: eumenitin-R from *Eumenes rubrofemoratus* [12], eumenitin-F from *Eumenes fraterculus* [12], and EpVP1 from *Eumenes pomiformis* [27]. In contrast to the case of mastoparans, eumenitin-type peptides have been found only in solitary wasp venoms, whereas social wasp venoms never contain this type of peptides. 

### 2.3. Protonectin

Orancis-protonectin (OdVP2) from *Orancistrocerus drewseni drewseni* is a dodecapeptide with an amidated C-terminus [25], and is closely related to protonectin, which was originally found in Brazilian social wasp venom. Its analogs are distributed in some other social wasp venoms [22]. Protonectins are rich in hydrophobic amino acid residues and exhibit potent hemolytic activity. Orancis-protonectin is the first example of the protonectin analog isolated from a solitary wasp venom. This peptide shows more potent hemolytic activity than that of mastoparan and moderate antimicrobial activity [25,26].

### 2.4. Decoralin

Decoralin from *Oreumenes decoratus* is a smaller peptide, with a length of only 11 amino acids without C-terminal amidation [11]. This sequence has the characteristic features of linear cationic α-helical peptides, rich in hydrophobic and basic amino acids with no disulfide bond, and accordingly, it can be predicted to adopt an amphipathic α-helix secondary structure, as indicated by circular dichroism [11]. In fact, on biological evaluation, decoralin exhibited a significant broad-spectrum antimicrobial activity and moderate mast cell degranulation and leishmanicidal activities, but showed virtually no hemolytic activity. A synthetic analog with C-terminal amidation showed much more potent activity in all the biological assays [11], and NMR experiments demonstrated its amphipathic α-helix secondary structure [28].

### 2.5. Anoplin

Anoplin was purified from the venom of the solitary spider wasp *Anoplius samariensis* as a minor peptide component [9], while the major component of this venom is α-pompilidotoxin (α-PMTX), a neurotoxin blocking sodium channel inactivation [20,29]. Anoplin consists of only 10 amino acids, and has typical chemical and biological characteristics of antimicrobial α-helical peptides. This is among the smallest antimicrobial peptides with an α-helical structure found from natural sources, which can be advantageous for structural modification, structure-activity relationships studies, and therapeutic applications as a new and useful antimicrobial agent. There are many studies along this line [30,31,32,33,34,35,36,37,38,39,40,41,42,43].

The antimicrobial α-helical peptides in solitary wasp venoms may have a key role in preventing potential microbial infection of the paralyzed preys during consumption by their larvae [9]. When injected into lepidopteran larvae, the α-helical peptides caused feeding disorder, which indicated that the α-helical peptides might function as non-specific neurotoxins or myotoxins by inducing cell lysis and venom-spreading factors by increasing cell permeability [44].

## 3. Physicochemical Properties and Secondary Structure

**Figure 1:**
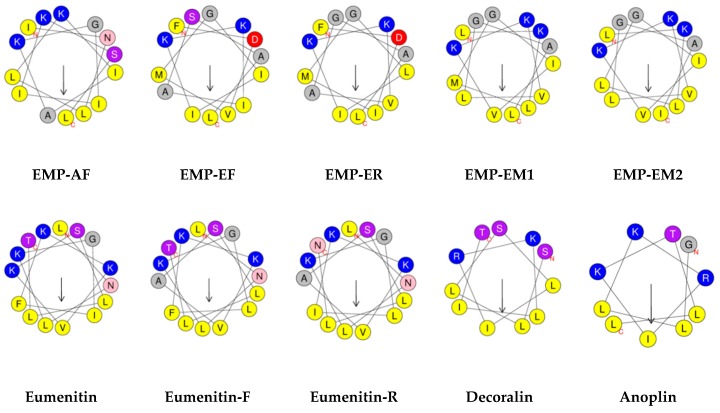
Helical wheel projection of antimicrobial α-helical peptides in solitary wasp venoms. In this view through the helix axis, the hydrophilic residues (D, N, K, S, T, R) are located on one side and the hydrophobic residues (F, I, L, M, V) on the other side of the helix. The arrow shows the hydrophobic moment (µ) vector. N: N-terminus; C: C-terminus.

Despite being produced by widely different organisms, antimicrobial peptides share similar structural patterns, which are known for their inter-dependency. The same applies for peptides found in solitary wasp venoms, which have been shown to be predominantly amphipathic, helical, and cationic at physiological pH. Some physicochemical properties have been investigated and shown to be important to understand structure-function relationships and to help designing new sequences with improved performance. Amongst solitary wasp venoms this tool has also been employed. Table 3 lists the physicochemical properties of antimicrobial peptides found in some solitary wasp venoms, which are briefly discussed below, and in Figure 1 helical wheel projections of these peptides are shown.

### 3.1. Sequence and Chain Length

To date, antimicrobial peptides from solitary wasp venoms have been found to be built almost exclusively by naturally occurring amino acids with a chain length ranging from 10 to 15 residues. Truncation studies with anoplin, a decapeptide, resulted in partial loss of the antimicrobial activity [30], suggesting that this chain length is close to the minimum required for significant activity. Truncation of three N-terminal residues inn EMP-AF totally abolished the biological activities [14]. Taken together, these results with anoplin and EMP-AF suggest that specific residues are more important than chain length, and that in such short chain peptides, features of some amino acid residues have a major importance. In solitary wasp venom peptides, anionic residues seldom appear, and among the cationic residues, Lys is much more frequent than Arg or His. Some of the apolar residues such as Trp, Pro, and Met seldom appear and polar uncharged residues such as Cys and Tyr are rare. On the other hand, bulky aliphatic residues such as Ile, Leu, and Val are ubiquitous, and similarly to what has been found for antimicrobial peptides in general they form the hydrophobic face [45,46]. 

### 3.2. N- and C-Termini

End effects are especially important for the structure and performance of short chain peptides [47]. The most frequent modification found with solitary wasp venom peptides is the amidation of the C-terminus, probably the most common posttranslational modification that occurs in a wide variety of peptides. Amidation involves oxidative decarboxylation of an additional glycine residue at the C-terminus. It prevents cleavage by carboxypeptidases, enables the accommodation of the otherwise negatively charged C-terminus in a nonpolar environment [48,49], and provides an extra hydrogen bond for the formation of α-helices [24]. The correlation between amidation and biological activity has been found to be an important feature, with carboxylated peptides showing significantly impaired activity [11,12,14,15]. 

### 3.3. Charge and Helix Macro-Dipole

The cationicity due to the net positive charge of solitary wasp venom peptides ranges from +1 to +4 (Table 3) and this feature has been considered essential for the activity of antimicrobial peptides, although the number of positive charges required for activity is case sensitive [48,50]. The action of cationic peptides on negatively charged pathogen membranes starts with the electrostatic interaction between them; hence, an increase in positive charge of peptides should increase microbial activity [48,51,52]. The first most comprehensive structure-function study carried out with a solitary wasp venom peptide and analogs was that by Ifrah and co-workers [30]. This work was recently further developed [33]. Although the impact of increasing the number of charged residues has not been specifically investigated to date, in the first work they showed that decreasing the number of positively charged residues either decrease antimicrobial activity or the selectivity, showing also an increase in the hemolytic activity. Increasing the net charge, the effect is position-dependent and most of the tested analogs showed increased hemolytic activity. In a more recent investigation [33], it was confirmed that increasing the charge and/or hydrophobicity improves antimicrobial activity, but also increases the hemolytic activity. With a different approach [36], the antimicrobial activity of anoplin was improved without compromising the therapeutic index by increasing the net charge, introducing Trp residues at specific positions, slightly modifying the retention times and increasing amphiphilicity. These findings are in good accordance with those by Dathe and co-workers [53] that showed that increasing the number of positive charges is followed by an increase in antimicrobial activity of magainin 2 analogs within certain limits. Above them, antimicrobial activity decreases and hemolytic activity appears. A minimum of charge around +2 has been suggested as required for antimicrobial peptide selectivity [54], because it facilitates initial interaction with microbial membranes, transitions in orientation (pore formation) and translocation of peptides to the cytoplasmic membrane, and helps displacing membrane-bound cations [48,54,55]. More recently, series of decoralin derivatives were studied for anticancer, antimicrobial, and antiplasmodial activities [56,57,58,59,60]. In the anticancer studies, it was observed that increasing the positive net charge favored the activity, although the amidated form of decoralin was among those with higher activity [56,59]. Concerning the antimicrobial activity, the works by Torres et al. [57,58] achieved lower hemolytic activity of the designed analogs in relation to amidated decoralin at equipotent antimicrobial levels. However, in the study of the antiplasmodial activity, while amidated decoralin was deprived of activity, three analogs showed potent activity and revealed that, for this activity, the net charge variation was not as important as it showed to be for the antimicrobial and anticancer activities [60].

Each peptide unit has a dipole moment of 3.5 D (1.155 × 10^−29^ Cm); in an α-helix the sum of individual residue dipoles, which are aligned almost parallel to the helix axis, builds up the helix dipole. The significant electric field of an α-helix is thus generated by the macro-dipole that runs from the negative pole at the C-terminus to the positive pole at the N-terminus. The strength of the field of this macro-dipole increases up to 10 Å helix length, which roughly corresponds to eight folded residues, and further elongation does not contribute significantly [61], although this field will be dependent on the solvent dielectric constant [62]. Considering the short chain length of solitary wasp venom peptides, the effects of the helix field have a major importance and represent a useful tool in the designing of new peptides since it contributes to the interaction with charged substrates in the long-range attraction, in facilitating the binding, and the suitable orientation in relation these substrates [61]. Nevertheless, the above-mentioned studies did not show differences in the activities that could be attributed to the helix macrodipole of the peptides. 

### 3.4. Helical Propensity and Helicity

The folding of an amino acid sequence into a helix is a cooperative process [63] characterized by the Zimm–Bragg parameters σ and s, respectively defined as nucleation factor and propagation constant [64,65]. Inner helical residues will form two hydrogen bonds, coil residues will form no hydrogen bonds, and end residues might form only one hydrogen bond to the third or fourth nearest neighboring residue. Each amino acid residue has its own conformational preferences, and in a sequence, the sum of these preferences leads to the stabilization or not of the α-helix [66], depending on chain length [65,67] and on the environment [68,69,70].

Helicity is a consequence of the primary sequence of amino acid residues [63,71] and of their interaction with the environment [68], which includes peptide hydrogen-bond formation and van der Waals and hydrophobic interactions, contributing to helix stabilization [66]. Helical structuring seems to be an important contributor to the antimicrobial action, since helical peptides are prevalent in the universe of antimicrobial peptides [45] and of solitary wasp venom peptides. Among factors that influence α-helix formation, there are (1) electrostatic interactions at the N-terminus, and at i, i +3 or i, i + 4 side chain interactions at the center of the chain [63] and (2) the hydrophobic interaction, as has been demonstrated by Blondelle and Houghten [72]. 

**Figure 2:**
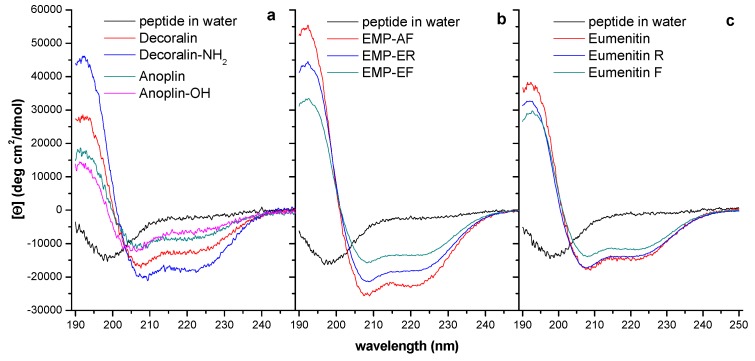
Examples of CD spectra obtained for solitary wasp venom peptides and analogs in 8-mM SDS solution. This micelle environment is frequently used as a mimetic of prokaryotic membranes due to its anionic character. (**a**) The shorter chain peptides, anoplin and decoralin, and their analogs: amidated forms display higher helical content. (**b**) The wild type amidated tetradecapeptides, EMP-AF, EMP-ER, and EMP-EF with +4 and +3 charges, respectively, share similar structural features with mastoparans and present higher helical content and higher hydrophobicity level. (**c**) The wild type carboxylated pentadecapeptides, eumenitin, eumenitin R, and eumenitin F with +3 charges and lower hydrophobicity level, show intermediate helicity level and correspond to the most selective solitary wasp venom peptides.

Amongst solitary wasp venom peptides and analogs, the helical content ranged from nearly 10 to 70%, more often around 50%, as determined in CD experiments (Table 3, Figure 2) in SDS micelles (8 mM). Although presenting preferential interaction with anionic media, in the presence of different membrane mimetic environments the helical fraction may vary, generally as a function of peptide hydrophobicity in electrically neutral media or as a function of peptide net charge in anionic environments. In aqueous or buffer media, solitary wasp venom peptides show random coil conformations [9,10,11,12,14,15]. Helical content influences peptide activity on negatively charged membranes, as is suggested by the examples in Table 3: there is a good correlation between the helical content and the antimicrobial activity among solitary wasp venom peptides. According to Dathe and Wieprecht [73] and Giangaspero and co-workers [46], higher helicity correlates with increased activity towards zwitterionic membranes. However, results obtained with model peptides suggested that helicity is more important to peptide activity on zwitterionic membranes than to permeabilization of the negatively charged ones [74]. Structural information on solitary wasp venom peptides is mainly based on CD data; to the best of our knowledge, NMR experiments were carried out just for EMP-AF [24] and decoralin [28]. Accumulating information obtained from the interaction of solitary wasp venom peptides with model membranes by NMR would help establishing a database with an increased confidence level.

### 3.5. Amphipathicity and Hydrophobic Moment

The most common structure among antimicrobial peptides and solitary wasp venom peptides, including analogs, is the amphipathic helix, ideal for interacting with equally amphipathic biomembranes. Amphipathicity reflects the possibility that an amino acid sequence has to form well-structured hydrophobic and hydrophilic domains in opposite faces. The hydrophobic moment represents a quantitative measurement of amphipathicity [75]. However, it assumes that 100% of the side-chains protrude perpendicular to the helix axis at regular 100° intervals, i.e., as an ideal α-helix. It could be more accurate for the short solitary wasp venom peptides if it were calculated based on NMR structural data. Nevertheless, a search among antimicrobial peptides showed that the hydrophobic moment ranges from 0.45 to 0.6 [45,46], while for solitary wasp venom peptides we found values ranging from around 0.2 to 0.4. Increasing the hydrophobic moment results in a significant increase in the permeabilizing and hemolytic activities of model peptide and magainin [52,76]. Pathak and co-workers [77] suggested that amphipathicity was more important than hydrophobicity or α-helical content in governing antimicrobial peptide activity. However, Dathe and co-workers, working with KLA model peptides designed to have individual parameter modifications, showed that increasing hydrophobic moment appears to have only modest effects on anionic bilayer permeabilizing efficiency, but for zwitterionic bilayers, where electrostatic peptide–lipid interactions are minimized, a higher hydrophobic moment results in a significant increase in permeabilizing activity [76]. It was suggested that an imperfect segregation of hydrophilic and hydrophobic groups of the peptide chain contributes to the bilayer disturbing abilities and thus to the antimicrobial activity, according to the proposed model of interfacial activity [78]. In this case, also an accumulation of NMR structural data would contribute to our knowledge of the real amphipaticity of peptides when bound to membranes or their models.

### 3.6. Hydrophobicity

In aqueous media hydrophobic units do not make hydrogen bond to water; this creates excluded volume regions where the absence of water enables attraction among apolar groups, originating hydrophobic interactions [79]. Hydrophobicity measures the peptide affinity for the membrane interior and is calculated as the average value of all peptide residues in the chain, according to different scales, but in the present work, according to the Eisenberg consensus scale [75]. Mean hydrophobicity levels among solitary wasp venom peptides range from as low as −0.11 to +0.14 and show selectivity for bacterial membranes (Table 3). Hydrophobicity is necessary for effective membrane permeabilization, however, increased levels favor interactions with acidic and zwitterionic vesicles, impairing the selectivity to bacterial membranes [45,54,80]. Blondelle and Houghten established good correlations between decreased hydrophobicity and increased antimicrobial activity and between decreased hydrophobicity and decreased hemolytic activity, working with de novo designed peptides [72]. 

From the physicochemical properties that influence the biological activities of antimicrobial peptides and solitary wasp venom peptides, mean hydrophobicity and charge have proved to be important modulating factors [81]. Dathe and co-workers showed that hydrophobicity at very low levels abolishes activity, at high levels enhances hemolysis, at too high levels cause aggregation or precipitation, and at reasonable low levels enhances Gram-negative bacterial specificity [52,73]. Besides discriminating lipid head groups [82] and the importance on the peptides adsorption to the lipid membrane, charged residues are shown by all atoms or coarse graining molecular dynamics simulations to play key role on the pore formation [83,84]. Simulations have also shown that hydrophobicity is important for peptide aggregation before pore formation [83,84] and, reinforced by X-ray experiments [85,86], hydrophobicity was demonstrated to influence the peptides embedding into the membrane core. The combination of higher charge (+4) and positive hydrophobicity (+0.051), as in the case of EMP-AF, increased the hemolytic activity, while anoplin and eumenitin R are good examples of the opposite effect. One of the great contributions of the structure-function studies with solitary wasp venom peptides is the fact that they highlight many possibilities of designing active antimicrobials considering short chains with lower toxicity.

## 4. Channel-Like Pore-Forming Properties 

Substances that are hemolytic or cytotoxic due to pore formation in membranes generally form non-selective pores that conduct ions, toxins, and metabolites that may produce progressive membrane depolarization and prevent both eukaryotic and prokaryotic cells to keep homeostasis [87,88]. The biological activities of solitary wasp venom peptides (antimicrobial, fungicidal, mast cell degranulating, hemolytic, and antiprotozoal activities) are often due to the adoption of an amphipathic α-helical secondary structure that inserts into the lipids of biological membranes. The membrane permeation induced by amphipathic α-helical peptides may occur by one of two general mechanisms: “barrel-stave”, that is, the formation of transmembrane pores; and the “carpet” mechanism, which causes membrane destruction or solubilization [89]. So far, anoplin, eumenitin, eumenitin-F, eumenitin-R, EMP-EF, and EMP-ER have been tested in artificial lipid bilayers, and the channel-like activity observed demonstrated that ion conduction through biological membranes must be important to their lytic activity against mammalian cells, but more importantly, against microorganisms.

### 4.1. Anoplin

Anoplin is the shortest known pore-forming peptide from a solitary wasp [9]. In experiments with planar lipid bilayers of asolectin, anoplin, which has an amidated C-terminus (ANP-NH_2_), formed pores in the artificial lipid bilayers, with channel-like activity [15]. 

The channel activity of ANP-NH_2_ starts 20–30 min after addition, under applied voltages preferentially above +100 mV. ANP-NH_2_ channels showed fluctuations of similar amplitudes (unitary currents) and occasionally, integer multiples of the unitary value [15]. Mean open times ranged from 39 to 45 ms, and open probabilities were higher at +100 mV, reaching 66%, with an average conductance of 50 pS (Table 4). The pores are essentially selective to cation, and the diameter of a pore was estimated to be 0.5–0.6 nm, considering KCl as conductive solution. 

The single channel conductance measurements with the carboxylated form of the peptide (ANP-OH) showed only small and rare transitions with maximum bilayer conductance of 40 pS at 100 mV potential. Under higher or lower holding potentials, transitions are even rarer and insignificant, showing that the amidation of the peptide C-terminus was crucial for channel-like activity in the anionic lipid bilayers of asolectin [15].

### 4.2. Eumenitin

The antimicrobial peptide eumenitin interacts preferentially with charged lipids, but incorporated channel-like pores in both charged (asolectin, negative) and zwitterionic (1,2-diphytanoyl-sn-glycero-3-phosphocholine or DPhPC) membranes. Interestingly, cholesterol addition to DPhPC membranes did not inhibit the binding of eumenitin to the membrane, as measured by the surface potential, but abolished the pore activity [16].

In asolectin bilayers, eumenitin at the concentration of 0.25 µM induced current fluctuations under a constant voltage pulse that corresponded to open (conducting) and closed (non-conducting) states. Under either +50 or −50 mV, the pore mean conductance was 118 pS (Table 4), and a second and less frequent conducting state was also observed, with conductance of 160 pS [16]. Using the same concentration of eumenitin in experiments with zwitterionic membranes (made of DPhPC), the observed pore activity was very different from the recordings in negatively charged membranes (asolectin). The mean conductance of single-channels in DPhPC bilayers at a +50 mV pulse was 61 pS, nearly half of asolectin’s membrane pore conductance (Table 3). Cholesterol addition to the DPhPC membranes abolished channel-like activity induced by eumenitin, even with increased voltage pulses (up to +100 mV) [16].

The eumenitin-induced pores presented a higher selectivity for cations over anions when tested in asolectin membranes, using KCl as conducting solution. Taking all information about eumenitin pores, Arcisio-Miranda and co-workers suggested that the formation of toroidal pores in membranes was the most adequate model to explain the biological activities of this peptide [16]. Matsuzaki and co-workers proposed this model for magainin 2 pores [89]. In this model, the interfacial peptide interaction induces the lipid monolayers to bend continuously through the pore, so that the water-filled core is lined by both inserted peptide and the lipid head groups, together forming well-defined pores. The use of negatively charged lipids, such as asolectin, would reduce the repulsive force due to the positive charge of the peptide (eumenitin has three positive charges, see Table 3). Thus, the ‘residual’ negative lipid charge could determine the cation selectivity observed for eumenitin, mainly in asolectin membranes [74].

### 4.3. Eumenitin-F and Eumenitin-R

Recently, four new linear cationic α-helical peptides from solitary wasp venoms were described [12]. Their sequences, physicochemical properties, channel incorporation and biological activities were studied to give a full profile of these peptides. Two of them have sequences related to eumenitin, thus named eumenitin-F and eumenitin–R. The other two are related to mastoparan, a class of peptide that was first found in social wasps [21], but has also been found in solitary eumenine wasps, the first being named EMP-AF [8]: EMP-EF and EMP-ER [12].

The two eumenitin peptides, eumenitin-F and eumenitin-R, were studied in mimetic lipid bilayers of asolectin obtained from GUVs (giant unilamellar vesicles). Using a 150-mM HCl bathing solution, eumenitin-F and eumenitin-R induced channel-like activity within 10 min of incubation (0.5–2 µM concentration, added to the *cis* side). Pore formation was observed in the presence of both peptides under positive and negative voltage pulses, and single-channel conductances were calculated. Eumenitin-F showed higher conductance levels, of 298.6 and 187.1 pS (−100 and +100 mV, respectively), while eumenitin-R channels had conductances of 82.5 and 118.8 pS at the same holding potentials (Table 4). Different conductance levels were detected in the pores formed, and were not double or triple of single channels conductance. Pores with conductances higher than 500 pS were recorded, indicating that clusters could be formed, and several units of the peptides organize to form bigger pores. Rectification was observed only in the eumenitin-F channels [12]. The above-mentioned eumenitin and anoplin pores presented similar behavior, as well as a social wasp peptide, HR-1, that has some similarities to mastoparan [90].

### 4.4. EMP-EF and EMP-ER

The two eumenine-mastoparan peptides, EMP-EF and EMP-ER (Table 1), that have high homology to mastoparan [21], and EMP-AF [8] presented channel-like activity in asolectin bilayers under positive and negative voltage pulses at concentrations ranging from 0.5 to 2 µM, added at the *cis* side. Single channel conductances formed by EMP-EF were 33.6 and 32.2 pS (−100 and +100 mV, respectively) lower than for EMP-ER channels (68.2 and 61.4) at the same holding potentials (Table 4). Double conductance levels were recorded, equivalent to the aperture of two single channels simultaneously, but no rectification was detected [12]. The pore conductance levels for EMP-EF and EMP-ER were equivalent to those for mastoparan HR-1, although the double conductance levels were recorded only in presence of EMP-EF and EMP-ER [12], but not with HR-1 [90]. The higher hydrophobicities of EMP-EF and EMP-ER when compared to HR-1 could account for the presence of double conductance levels in the recordings with the eumenine mastoparan peptides. 

According to Rangel and co-workers [12], the new eumenitins (eumenitin-F and eumenitin-R) and mastoparan peptides’ (EMP-EF and EMP-ER) pore-like activity and other characteristics, such as the length (shorter than bilayer thickness) and bulky residues, favor the toroidal pore model. By this model, the pore is described as a complex made of lipid molecules, predominantly, and peptide molecules that insert into the bilayer, inducing its destabilization [91,92]. The toroidal pore model was also proposed for the pores induced by the peptide eumenitin [16], and due to the high homology of these peptides it is the best model to explain the electrophysiology results so far.

It is interesting to highlight that the conformational and pore-forming activity of the peptides above were investigated predominately in asolectin bilayers, which due to its anionic character mimic the cytoplasmic membrane of bacteria. This phospholipid mixture has an approximate composition of 23.5% phosphatidylcholine, 20% phosphatidylethanolamine, and 14% inositol phosphatides (other components are 39.5% other phospholipids, lipids, and carbohydrates, and 2% triglycerides, tocopherols, and sterols). It holds some similarities to the lipid composition of rat mast cells; the phospholipids amount roughly to 50% of the total lipids. From these, phosphatidylcholine represents 30%, phosphatidylethanolamine 27%, sphingomyelin 20%, and phosphatidylserine and phosphatidylinositol 16%. An important difference lies in cholesterol, which represents around 20% of the total lipid content in rat mast cell membranes, while in asolectin sterols represent less than 0.3% [93]. In relation to sterols and the general anionic character, this bilayer can also be considered a mimetic of microbial membranes. Thus, the behavior of the eumenine peptides can be reasonably well modeled and their mechanism of action understood with the use of asolectin bilayers. Furthermore, when other lipids such as DPhPC and DPhPC with cholesterol were used to form planar bilayers with zwitterionic character, the pore-forming activity of the eumenitin peptide was reduced or even abolished [16]. This may be due to the positive net charge of the peptides (Table 3), and the lipid charge that may favor or diminish the interactions and pore-forming capability of these cationic peptides. Furthermore, cholesterol, which is only present in eukaryotic cells, changes the interactions of cationic peptides with the membrane [94]. Cholesterol also affects the fluidity and the dipole potential of phospholipid membranes [95].

**Figure 3:**
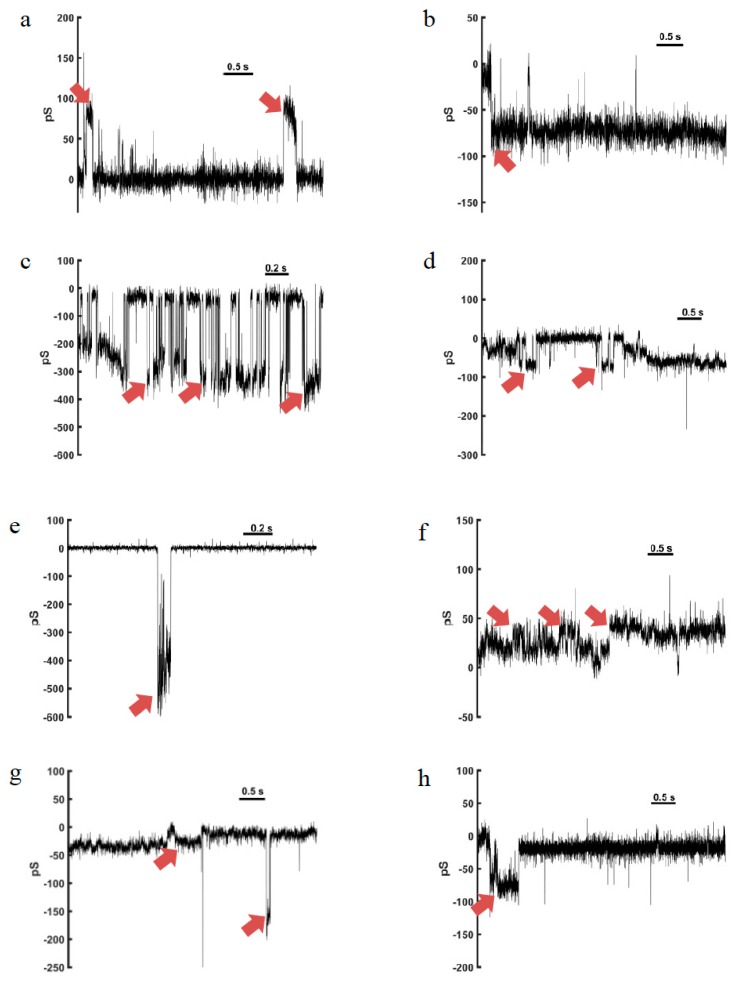
Representative recordings of single channel incorporation of solitary wasp venom peptides in asolectin bilayers. Eumetin-F (**a**–**c**) and eumenitin–R (**d**,**e**), both with carboxylated C-terminus, formed pores with low (<100 pS) and high conductance (>400 pS) levels. EMP-EF (**f**,**g**) and EMP-ER (**h**), however, presented higher hemolytic activity due to the amidated C-terminus, but incorporated channels of low conductance. Arrows indicate channel apertures.

The amidated C-terminus was favorable to the hemolytic and mast cell degranulating properties of the peptides, as observed for EMP-AF [8], EMP-EF, and EMP-ER [12] when compared with eumenitins [10,12]. However, the high conductance pores in artificial bilayers were only recorded with peptides that have carboxylated C-terminus, eumenitin-F, and eumenitin-R (Figure 3) [12]. 

## 5. Concluding Remarks

Our studies on solitary wasps surveying bioactive components in their venoms revealed that antimicrobial peptides are contained in many wasp venoms, mostly in eumenine wasp venoms. Their chemical and biological characteristics are typical for linear cationic α-helical peptides. Physicochemical properties and the pore-forming activity of these peptides were investigated in detail, and the results can be useful for investigation of the structure-activity relationship and mechanism of action.

Antimicrobial α-helical peptides are widely distributed in arthropod venoms e.g., scorpion and spider venoms. They may function not only in preventing the prey from microbial infection during long-time storage, but also in potentiating venom toxicity by disturbing excitable membranes [5,6,7]. Similarly, the solitary wasp venom peptides can act as antimicrobials against microbial infection [9] and as a venom toxicity potentiator [44].

The simple structure (consisting of only 10–15 amino acids without disulfide bonds) of the solitary wasp venom peptides is advantageous for chemical modification and structure-activity relationship studies. Furthermore, some of these peptides show only weak or virtually no hemolytic activity, which is another advantage especially for medical applications and development. In particular, anoplin and decoralin have lengths of only 10 and 11 amino acids, respectively, with virtually no hemolytic activity. There are many studies based on these peptides towards developing new antibiotic and anticancer agents [30,31,32,33,34,35,36,37,38,39,40,41,42,43,57,58,59,60].

## Figures and Tables

**Table 1:** Antimicrobial and α-helical peptides in solitary wasp venoms. EMP: eumenine mastoparan.

Peptide	Sequence	Peptide	Sequence
EMP-AF	INLLKIAKGIIKSL-NH_2_	Eumenitin	LNLKGIFKKVKSLLT
EMP-EF	FDVMGIIKKIASAL-NH_2_	Eumenitin-F	LNLKGLFKKVASLLT
EMP-ER	FDIMGLIKKVAGAL-NH_2_	Eumenitin-R	LNLKGLIKKVASLLN
EMP-EM1	LKLMGIVKKVLGAL-NH_2_	EpVP1	INLKGLIKKVASLLT
EMP-EM2	LKLLGIVKKVLGAI-NH_2_	Decoralin	SLLSLIRKLIT
EpVP2a	FDLLGLVKKVASAL-NH_2_	OdVP3	KDLHTVVSAILQAL-NH_2_
EpVP2b	FDLLGLVKSVVSAL-NH_2_	EMP-OD (OdVP1)	GRILSFIKGLAEHL-NH_2_
Anoplin	GLLKRIKTLL-NH_2_	Orancis-Protonectin (OdVP2)	ILGIITSLLKSL-NH_2_

**Table 2:** Selected biological activities of antimicrobial and α-helical peptides in solitary wasp venoms.

Peptides	Antimicrobial Activity (MIC, μM)			Hemolysis (EC_50_, μM)	Degranulation (EC_50_, μM)	References
	*S. aureus*	*E. coli*	*C. albicans*			
**EMP-AF**	3	33	-	50	30	[8,14]
**EMP-EF**	30	30	7.5	181	60	[12]
**EMP-ER**	30	30	7.5	200	70	[12]
**EMP-EM1**	7	7	>60	na	70	[13]
**EMP-EM2**	3	3	>60	na	60	[13]
**Eumenitin**	6	6	-	na	70	[10]
**Eumenitin-F**	>60	30	7.5	353	na	[12]
**Eumenitin-R**	60	30	7.5	530	na	[12]
**Decoralin**	40	80	40	na	na	[11]
**Anoplin**	4	43	-	na	30	[9]

MIC: minimum inhibitory concentration; EC_50_: effective concentration that produces 50% of the maximum effect; na: no activity or virtually no activity; *S. aureus*: *Staphylococcus aureus* ATCC 6538 or ATCC 25923 (Gram-positive bacteria; *E. coli*: *Escherichia coli* CCT 6538 or ATCC 25922 (Gram-negative bacteria); *C. albicans*: *Candida albicans* (UMP) (Yeast); Hemolysis: human or mouse erythrocytes; Degranulation: rat peritoneal mast cells or RBL-2H3 cells.

**Table 3:** Physicochemical parameters and α-helical content of antimicrobial peptides in solitary wasp venom in different environments.

Peptides	*N*	Q	C-Term	<H>	µ	*f_H_*			References
						TFE	SDS	PC	
**EMP-AF**	14	+4	amide	0.051	0.342	0.55	0.72	0.16	[14]
**EMP-EF**	14	+2	amide	0.115	0.279	0.41	0.44	nd	[12]
**EMP-ER**	14	+2	amide	0.131	0.251	0.53	0.59	nd	[12]
**EMP-EM1**	14	+4	amide	0.104	0.258	0.31	0.33	0.11	[13]
**EMP-EM2**	14	+4	amide	0.138	0.278	0.37	0.41	< 0.03	[13]
**Eumenitin**	15	+3	carboxyl	0.002	0.265	0.43	0.50	0.07/0.11	[10]
**Eumenitin-F**	15	+3	carboxyl	-	0.256	0.34	0.44	nd	[12]
**Eumenitin-R**	15	+3	carboxyl	-	0.281	0.43	0.48	nd	[12]
**Decoralin**	11	+3	carboxyl	0.028	0.393	0.38	0.50	rc	[11]
**Anoplin**	10	+4	amide	-	0.366	0.24	0.26	rc	[9]

***N***: number of residues; **Q**: net charge; **C-term**: C-terminus; **<H>**: mean hydrophobicity; **μ**: hydrophobic moment; ***f*_H_**: α-helix fraction, 40% TFE, 8 mM SDS, 380 µM PC; nd: non-determined; rc: random coil.

**Table 4:** Conductances of pores induced by solitary wasp venom peptides in anionic (asolectin) or zwitterionic (DPhPC and DPhPC-cholesterol) bilayers, according to the V_hold_ (mean and standard error of mean, minimum of three different experiments).

Peptides	Lipid	V_hold_ (mV)	Conductance (pS)	SEM	References
**Eumenitin**	asolectin	−50	118.0/160.0	3.67/7.07	[16]
	asolectin	+50	118.0/160.0	3.67/7.07	[16]
	DPhPC	+50	61.13	7.57	[16]
	DPhPC-cholesterol	−100	None	-	[16]
	DPhPC-cholesterol	+100	None	-	[16]
**Eumenitin-R**	asolectin	−100	82.5	17.1	[12]
	asolectin	+100	118.8	44	[12]
**Eumenitin-F**	asolectin	−100	298.6	51	[12]
	asolectin	+100	187.1	67.7	[12]
**EMP-ER**	asolectin	−100	68.2	4	[12]
	asolectin	+100	61.4	3.7	[12]
**EMP-EF**	asolectin	−100	33.6	8.9	[12]
	asolectin	+100	32.2	6.9	[12]
**Anoplin**	asolectin	+100	50.7	3.8	[15]
	asolectin	+130	58.4	2.7	[15]
	asolectin	+150	66.8	6.7	[15]

DPhPC: 1,2-diphytanoyl-sn-glycero-3-phosphocholine; V_hold_: clamping voltage in milivolts; pS: picosemens.

## Abbreviations

EMP	eumenine mastoparan
PMTX	pompilidotoxin
HPLC	high performance liquid chromatography
CD	circular dichroism
TFE	2,2,2-trifluoroethanol
SDS	sodium dodecylsulfate
NMR	nuclear magnetic resonance
KLA	lysine-leucine-alanine (amino acid sequence)
DPhPC	1,2-diphytanoyl-sn-glycero-3-phosphocholine
